# The Effect of Ventilation with Individualized Positive End-Expiratory Pressure on Postoperative Atelectasis in Patients Undergoing Robot-Assisted Radical Prostatectomy: A Randomized Controlled Trial

**DOI:** 10.3390/jcm10040850

**Published:** 2021-02-19

**Authors:** Hyun-Kyu Yoon, Bo Rim Kim, Susie Yoon, Young Hyun Jeong, Ja Hyeon Ku, Won Ho Kim

**Affiliations:** 1 Department of Anesthesiology and Pain Medicine, Seoul National University Hospital, Seoul National University College of Medicine, #101 Daehak-ro, Jongno-gu, Seoul 03080, Korea; hyunkyu18@gmail.com (H.-K.Y.); petitbelle22@naver.com (B.R.K.); susiey87@gmail.com (S.Y.); yhhh1130@naver.com (Y.H.J.); 2 Department of Urology, Seoul National University Hospital, Seoul National University College of Medicine, #101 Daehak-ro, Jongno-gu, Seoul 03080, Korea; kukuro70@snu.ac.kr

**Keywords:** lung compliance, positive end-expiratory pressure, prostatectomy, pulmonary atelectasis, ultrasonography

## Abstract

For patients undergoing robot-assisted radical prostatectomy, the pneumoperitoneum with a steep Trendelenburg position could worsen intraoperative respiratory mechanics and result in postoperative atelectasis. We investigated the effects of individualized positive end-expiratory pressure (PEEP) on postoperative atelectasis, evaluated using lung ultrasonography. Sixty patients undergoing robot-assisted radical prostatectomy were randomly allocated into two groups. Individualized groups (*n* = 30) received individualized PEEP determined by a decremental PEEP trial using 20 to 7 cm H_2_O, aiming at maximizing respiratory compliance, whereas standardized groups (*n* = 30) received a standardized PEEP of 7 cm H_2_O during the pneumoperitoneum. Ultrasound examination was performed on 12 sections of thorax, and the lung ultrasound score was measured as 0–3 by considering the number of B lines and the degree of subpleural consolidation. The primary outcome was the difference between the lung ultrasound scores measured before anesthesia induction and just after extubation in the operating room. An increase in the difference means the development of atelectasis. The optimal PEEP in the individualized group was determined as the median (interquartile range) 14 (12–18) cm H_2_O. Compared with the standardized group, the difference in the lung ultrasound scores was significantly smaller in the individualized group (−0.5 ± 2.7 vs. 6.0 ± 2.9, mean difference −6.53, 95% confidence interval (−8.00 to −5.07), *p* < 0.001), which means that individualized PEEP was effective to reduce atelectasis. The lung ultrasound score measured after surgery was significantly lower in the individualized group than the standardized group (8.1 ± 5.7 vs. 12.2 ± 4.2, mean difference −4.13, 95% confidence interval (−6.74 to −1.53), *p* = 0.002). However, the arterial partial pressure of the oxygen/fraction of inspired oxygen levels during the surgery showed no significant time-group interaction between the two groups in repeated-measures analysis of variance (*p* = 0.145). The incidence of a composite of postoperative respiratory complications was comparable between the two groups. Individualized PEEP determined by maximal respiratory compliance during the pneumoperitoneum and steep Trendelenburg position significantly reduced postoperative atelectasis, as evaluated using lung ultrasonography. However, the clinical significance of this finding should be evaluated by a larger clinical trial.

## 1. Introduction

Radical prostatectomy is considered the treatment of choice for a localized prostate cancer [1]. Robot-assisted radical prostatectomy has shown more favorable perioperative outcomes than open radical prostatectomy [2]. For robot-assisted surgery, pneumoperitoneum and a steep Trendelenburg position are required for surgical access [3]. However, the pneumoperitoneum worsens respiratory function during surgery, particularly by atelectasis formation [4,5], which could be further aggravated by the steep Trendelenburg positioning [6,7]. As postoperative atelectasis might adversely affect patient recovery after surgery [8], it is imperative to prevent the development of atelectasis during the pneumoperitoneum.

One of the main components of the intraoperative lung-protective ventilator strategy is the application of positive end-expiratory pressure (PEEP) [9]. Although there is accumulating evidence that the levels of PEEP should be individualized during the surgery, rather than setting a uniformly fixed PEEP, the optimal level of PEEP and how to individualize PEEP remain arguable [10]. According to previous studies [11,12,13], determining individualized PEEP according to maximal respiratory compliance is simple and shows promising results. 

As a bedside diagnostic tool, lung ultrasound has been suggested for the diagnosis of perioperative atelectasis [14]. According to previous studies [15,16], ultrasonographic features indicating aeration loss such as B lines and subpleural consolidation are significantly correlated with the amount of pulmonary atelectasis. In subsequent clinical trials, lung ultrasound successfully identified perioperative atelectasis [17,18,19,20].

Therefore, we designed a randomized controlled trial to evaluate the effects of ventilation with individualized PEEP, aiming to maximize respiratory compliance, on postoperative atelectasis in patients undergoing robot-assisted radical prostatectomy. We hypothesized that individualized PEEP would reduce postoperative atelectasis and improve respiratory mechanics. We evaluated this hypothesis by comparing the lung ultrasound scores measured before and after general anesthesia. As secondary outcomes, the incidence of a composite of postoperative respiratory complications and intraoperative respiratory parameters was compared between the groups receiving individualized or standardized PEEP.

## 2. Materials and Methods

### 2.1. Trial Design and Participants

This single-center prospective randomized controlled trial was approved by the Institutional Review Board of Seoul National University Hospital (1908-022-1053), South Korea. Before patient enrollment, the study protocol was registered at Clinicaltrials.gov (NCT04085146, Principal investigator: W.H.K., Date of registration: 11 September 2019). This study was performed under the Good Clinical Practice Guidelines and adhered to the applicable Consolidated Standards of Reporting Trials (CONSORT) guidelines. Written informed consent was obtained from all patients before enrollment. Patients aged ≥20 years and scheduled for elective robot-assisted radical prostatectomy between November 2019 and May 2020 were enrolled. Patients with the following features were excluded: (1) American Society of Anesthesiologists physical status classification III or more, (2) moderate or severe obstructive or restrictive pulmonary disease indicated by a preoperative pulmonary function test, (3) history of heart failure, unstable angina, and increased intracranial pressure, and (4) history of pneumothorax or presence of giant bullae. 

Block randomization was performed by an investigator who was blinded to the present study. The patients were randomly allocated with a 1:1 ratio in blocks of four to one of the two groups: the group receiving individualized PEEP during pneumoperitoneum (individualized group) and the group receiving a standardized PEEP during pneumoperitoneum (standardized group). The patients, surgeons, and investigators did not know which group the patients had been assigned to. The allocation order, which was concealed in an opaque envelope, was disclosed just before anesthesia induction.

### 2.2. Anesthesia and Study Interventions

After they entered the operating room without any premedication, all participants were monitored using standard monitors, including non-invasive blood pressure, pulse oximetry, and three-lead electrocardiography. Preoxygenation was performed for 3 min with a vital capacity maneuver, providing 8 L/min of oxygen with a fraction of inspired oxygen of 1.0. Then, anesthesia was induced by using a target-controlled infusion of 4 μg/mL of propofol and 4 ng/mL of remifentanil. After confirmation of the loss of consciousness, 0.6–0.8 mg/kg of rocuronium was administered to facilitate endotracheal intubation, and the radial artery was catheterized for continuous blood pressure monitoring. Mechanical ventilation was initiated via volume-controlled ventilation, and ventilator parameters were initially set at a fraction of inspiratory oxygen (FiO_2_) of 0.4, a tidal volume of 6 mL/kg of predictive body weight, a PEEP of 7 cm H_2_O, and an inspiratory-to-expiratory ratio of 1:2. Anesthesia was maintained according to our institutional protocol by maintaining the bispectral index at 40–60 and the mean arterial pressure at 60–90 mmHg.

**Figure 1:**
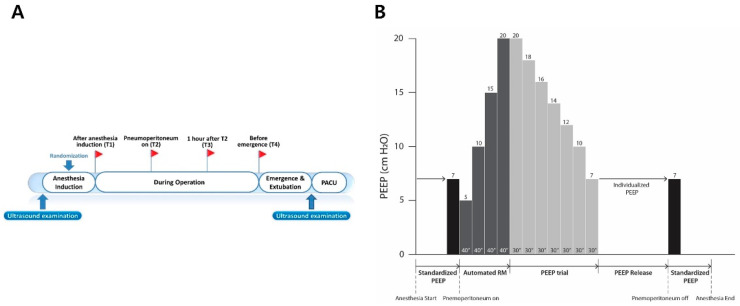
Study protocol. (**A**) Four predefined time points (T1–T4) when intraoperative ventilatory parameters were recorded and arterial blood gas analysis was performed and two time points of ultrasound examination. (**B**) The protocol of recruitment maneuver and decremental PEEP trial in the individualized group. These protocols were performed just before T2. This recruitment maneuver was also performed in the standardized group. RM = recruitment maneuver.

After pneumoperitoneum with a steep Trendelenburg position was achieved, ventilator mode was changed to pressure-controlled ventilation during alveolar recruitment in both groups (Figure 1). Alveolar recruitment was achieved by increasing the PEEP of 5 cm H_2_O to 20 cm H_2_O for 40 s at each level of PEEP while maintaining the pressure-controlled ventilation with the driving pressure of 20 cm H_2_O. This protocol of recruitment was performed by the automated built-in function of our anesthesia ventilator (Aisys^®^ Care Station 2; GE Healthcare, Madison, WI, USA). Alveolar recruitment was performed once every hour after the initial alveolar recruitment in both groups. After alveolar recruitments were performed, ventilator settings were adjusted depending on the allocation group.

For the individualized group, individualized PEEP was determined by a decremental PEEP trial using the automated procedure of the anesthesia ventilator (Aisys^®^), which adjusted the PEEP level by decreasing the level of PEEP from 20 to 7 cm H_2_O after alveolar recruitment. The individualized PEEP was determined as the lowest PEEP to achieve the best dynamic compliance during the decremental PEEP trial, and this level of PEEP was maintained during pneumoperitoneum. For the standardized group, a fixed PEEP of 7 cm H_2_O, which was initially set after anesthesia induction, was maintained during pneumoperitoneum. In both groups, if the peak inspiratory pressure exceeded 30 cm H_2_O above PEEP, the ventilation mode could be switched to the pressure-controlled mode from the initial volume-controlled mode. Additionally, when pulse oxygen saturation (SpO_2_) fell below 95% intraoperatively in both groups, we increased the FiO_2_ stepwise by 0.1 after alveolar recruitment using stepwise increases of PEEP from 5 to 20 cm H_2_O. When SpO_2_ still fell below 95% under the FiO_2_ of 1.0, the PEEP was increased stepwise by 2 cm H_2_O after alveolar recruitment. 

**Figure 2:**
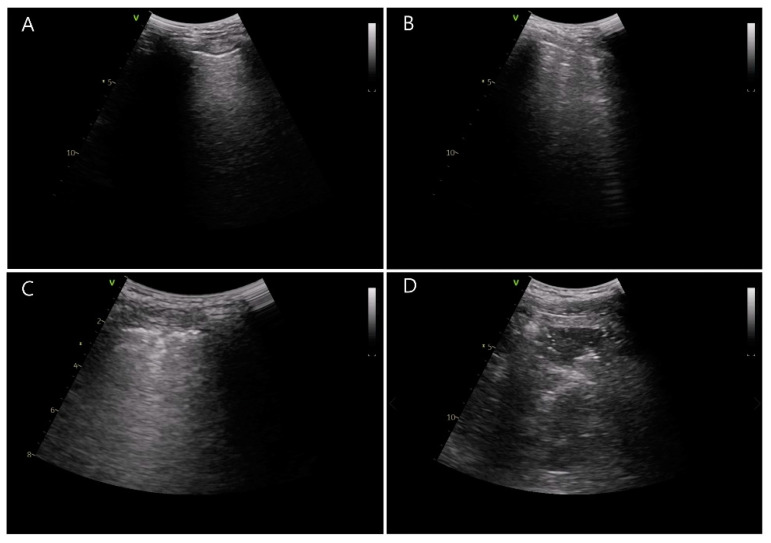
Lung ultrasound scores determined by the number of B lines and subpleural consolidation. (**A**) Normal aeration with 0–2 B lines, score = 0, (**B**) a small loss of aeration with ≥3 B lines, score = 1, (**C**) a moderate loss of aeration with multiple coalescent B lines or small subpleural consolidation, score = 2, and (**D**) a severe loss of aeration with consolidation or large subpleural consolidation, score = 3.

By using ultrasound (Vivid-q; GE Healthcare, Wauwatosa, WI, USA), atelectasis was examined by two trained investigators blinded to the group allocation. Ultrasound examination was conducted at two predefined time points: before the induction of general anesthesia and just after extubation in the operating room. Based on a previous study [21], the thorax was divided into 12 sections and the lung ultrasound score was evaluated as 0–3 by considering the count of B lines and the degree of subpleural consolidation (Figure 2) [16]. These scores were summed up from six quadrants of each hemithorax. The summed score of 0 to 2 is considered to be a normal reference value interval. As shown in Figure 2, a score of 0 represents normal aeration with 0–2 B lines, a score of 1 represents a small loss of aeration with ≥3 B lines, a score of 2 represents a moderate loss of aeration with multiple coalescent B lines or small subpleural consolidation, and a score of 3 represents a severe loss of aeration with consolidation or large subpleural consolidation.

### 2.3. Study Outcomes

The primary outcome of the present study was the absolute difference in the two lung ultrasound scores measured at the pre-induction period and just after extubation in the operating room. Secondary outcomes included the absolute values of the lung ultrasound score measured just after extubation in the operating room and the incidence of intraoperative desaturation defined as SpO_2_ of 95% or less. The incidence of a composite of postoperative respiratory complications and a composite of other complications was also compared. Respiratory complications included hypoxemia defined as SpO_2_ of 95% or less, laryngospasm, bronchospasm, pneumonia, pulmonary infiltration, pleural effusion, pulmonary edema, aspiration pneumonia, development of acute respiratory distress syndrome, atelectasis detected in chest radiography, and pneumothorax. A composite of other postoperative complications included surgical wound infection, acute kidney injury, surgical re-intervention, surgical wound dehiscence, length of hospital stays, admission to the intensive care unit, and incidence of subcutaneous emphysema. Intraoperative ventilatory parameters, including peak inspiratory pressure and dynamic and static compliance, were recorded at four predefined time points: (1) after anesthesia induction (T1), (2) after achievement of pneumoperitoneum under the steep Trendelenburg position (T2), (3) 1 h after T2 (T3), and (4) just before extubation in the operating room (T4). The driving pressure was calculated at these four time points by subtracting PEEP from plateau pressure. In the individualized group, T2 was also after individualized PEEP was set. Arterial blood gas was analyzed at the same time points, and arterial partial pressure of oxygen/fraction of inspired oxygen (PaO_2_/FiO_2_), arterial partial pressure of carbon dioxide (PaCO_2_), pH, bicarbonate (HCO_3−_), and arterial blood saturation (SaO_2_) were also recorded. 

As no previous studies have compared lung ultrasound scores in patients undergoing robot-assisted radical prostatectomy, the sample size was calculated using the data from our pilot study. When these patients received a standardized PEEP of 7 cm H_2_O during the pneumoperitoneum, the mean and standard deviation (SD) of the lung ultrasound score measured just after extubation in the operating room were 18 and 7.5, respectively. When we assumed that applying individualized PEEP to these patients during the pneumoperitoneum would decrease the postoperative lung ultrasound score to 12 with the same SD, given an alpha error of 0.05 and a power of 80%, 26 patients were required per group. Considering a dropout rate of 10%, a total of 60 patients were required.

### 2.4. Statistical Analysis

SPSS software version 25.0 (IBM Corp., Armonk, NY, USA) and R software version 3.6.1 (R Development Core Team, Vienna, Austria) were used to analyze the data. The normal distribution of our data was determined using the Shapiro–Wilk test and a visual inspection of the histogram and the quintile-quintile plot. Continuous variables were compared using the Student’s *t*-test or the Mann–Whitney *U* test, depending on the normality of the data. Discrete variables were compared using the chi-squared test or Fischer’s exact test according to their expected counts. For the comparison of baseline characteristics, we calculated standardized differences between groups. For all analyses, a *p*-value < 0.05 was considered to indicate a statistical significance. Additionally, intraoperative ventilatory parameters and the results of arterial blood gas analysis were compared using repeated-measures analysis of variance (ANOVA), which was followed by post-hoc analysis. Bonferroni correction was used to adjust for the increased alpha error by the multiple comparisons.

## 3. Results

**Figure 3:**
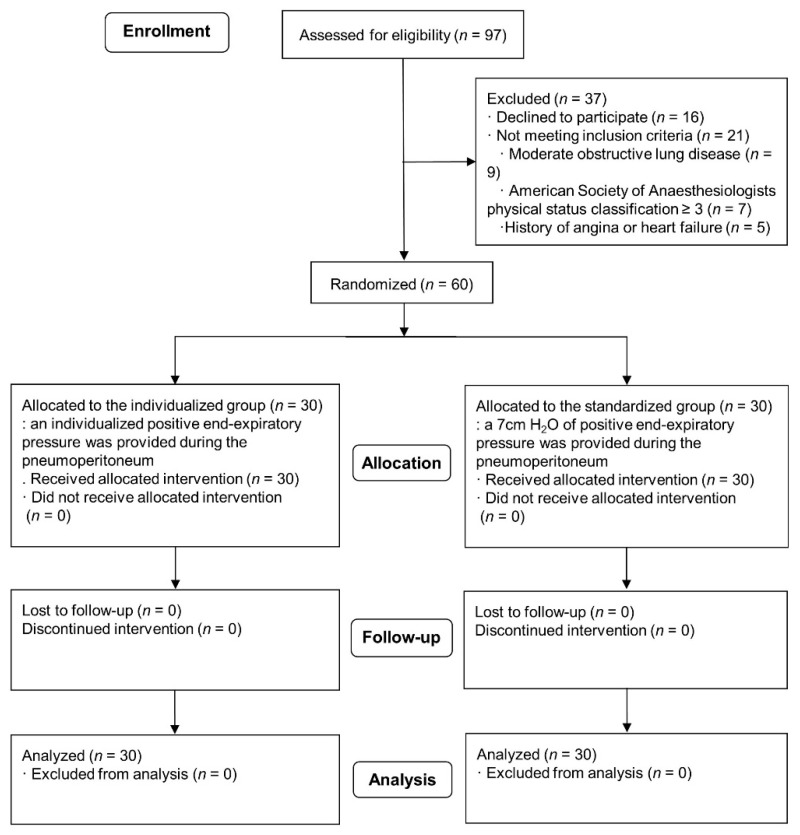
Consolidated Standards of Reporting Trials (CONSORT) flowchart.

Of the 97 patients eligible for the study, 37 were excluded. Sixteen patients refused to participate in this study, and 21 patients did not meet our inclusion criteria. The remaining 60 patients were enrolled in the study (Figure 3). The demographics and baseline characteristics were compared between the two groups (Table 1). Intraoperative anesthesia-related variables are compared in Table 2.

The FiO_2_ was adjusted up to 0.6 due to desaturation during surgery in both groups except for a single case of temporary using 1.0. We did not experience any PEEP adjustments due to desaturation (<95%) despite a FiO_2_ of 1.0 during surgery. There was no significant difference in the incidence of rescue recruitment maneuver of increasing FiO_2_ or the incidence of intraoperative desaturation between the individualized and standardized groups (3.3% vs. 13.3%; *p* = 0.353). There was no case of desaturation (<95%) despite providing the oxygen with a FiO_2_ of 1.0.

The optimal PEEP in the individualized group was determined as the median (interquartile range) 14 (12–18) cm H_2_O. Our primary outcome of the absolute difference in the lung ultrasound scores of the individualized group was significantly smaller than that in the standardized group (individualized group: mean ± SD, −0.5 ± 2.7 vs. standardized group: 6.0 ± 2.9; *p* < 0.001) (Table 3). Compared with the standardized group, the absolute value of the lung ultrasound score measured just after extubation was significantly lower in the individualized group (8.1 ± 5.7 vs. 12.2 ± 4.2; *p* = 0.002). The incidence of use of vasopressor, including ephedrine and phenylephrine (90.0% vs. 56.7%; *p* = 0.004), as well as the amount of crystalloid administration was significantly higher in the individualized group than the standardized group (1270.0 ± 486.6 vs. 838.3 ± 347.8 mL; *p* < 0.001) (Table 2).

**Figure 4:**
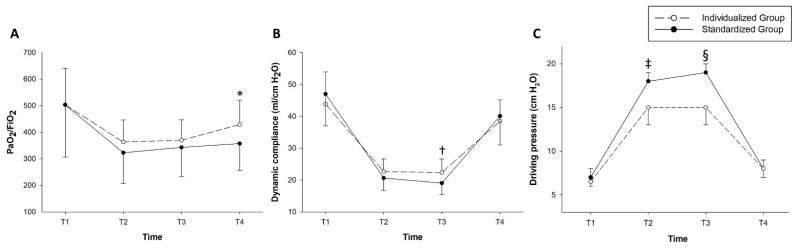
Intraoperative changes in the ventilator-related parameters. (**A**) shows arterial partial pressure of oxygen/fraction of inspired oxygen (PaO_2_/FiO_2_), (**B**) shows dynamic respiratory compliance, and (**C**) shows driving pressure. *: *p* = 0.005, †: *p* = 0.002, and ‡: *p* = 0.004 and §: *p* < 0.001, respectively. Four time points were predefined for recording intraoperative ventilatory parameters and performing arterial blood gas analysis as follows: T1 = after anesthesia induction, T2 = after achieving the pneumoperitoneum under the steep Trendelenburg position, T3 = 1 h after T2, and T4 = just before extubation in the operating room.

Repeated-measures ANOVA showed that PaO_2_/FiO_2_ levels during the surgery showed no significant time-group interaction between the groups (*p* = 0.145) (Figure 4).

The time-group interactions in the dynamic compliance and driving pressure were significant (*p* = 0.006 and *p* < 0.001, respectively). Post-hoc analysis with Bonferroni correction showed that the dynamic compliance at T3 was significantly higher in the individualized group than in the standardized group (22.3 ± 4.3 vs. 19.0 ± 3.5; *p* = 0.002). The driving pressure at T2 and T3 were also significantly lower in the individualized group than in the standardized group (T2: 15.0 (13.0–17.5) vs. 18.0 (15.5–19.5), *p* = 0.004; T3: 15.0 (13.0–17.5) vs. 19.0 (17.0–20.0), *p* < 0.001, respectively) (Figure 4). Ventilator-related parameters and intraoperative arterial blood gas analysis were compared between the two groups (Supplementary Table S1).

Regarding the composite of postoperative respiratory complications, the overall incidence was 41.7% (25/60) and there was no significant difference between the two groups (*p* = 0.190) (Table 3). The incidence of other postoperative complications, including anastomotic site leakage, acute kidney injury, and angina, was comparable between the two groups (*p* = 0.999).

## 4. Discussion

This randomized controlled trial investigated the effects of mechanical ventilation with individualized PEEP on postoperative atelectasis in patients undergoing robot-assisted radical prostatectomy. The optimal PEEP that maximizes respiratory compliance was determined by a decremental PEEP trial. We demonstrated that individualized PEEP significantly reduced postoperative atelectasis, as assessed using ultrasonography. However, there was no significant difference in the incidence of a composite of postoperative respiratory complications. Our study was not powered to detect differences in the composite of complications.

Evidence has been accumulated for the use of point-of-care lung ultrasound [22]. Although computed tomography is considered the gold standard for the diagnosis of atelectasis, its intraoperative use has several disadvantages compared to that of ultrasonography, which is simple, non-invasive, and free of irradiation [15]. Previous studies have validated the use of perioperative lung ultrasonography to detect atelectasis [17,19,20]. These studies investigated the effects of intraoperative alveolar recruitment in infants [20] and pediatric patients [17,19]. Another recent study investigated the effects of PEEP plus alveolar recruitment on atelectasis by the lung ultrasound score, which grades the severity of atelectasis according to ultrasound findings [18]. Although a previous study reported the effect of individualized PEEP plus alveolar recruitment on PaO_2_/FiO_2_, individualized PEEP was titrated using electrical impedance tomography and lung ultrasound was not used to evaluate atelectasis [23]. To the best of our knowledge, no study has examined the effects of individualized PEEP on postoperative atelectasis, as measured by ultrasonography.

Postoperative atelectasis develops in most surgical patients undergoing general anesthesia [24,25]. Atelectasis could result in decreased pulmonary compliance, impaired oxygenation, and increased pulmonary vascular resistance [8]. Additionally, the amount of atelectasis correlates well with impairment of gas exchange [26]. The combination of pneumoperitoneum with a steep Trendelenburg position induces cephalad movement of the diaphragm, leading to significant adverse physiologic changes, such as decreased lung compliance and functional residual capacity [27,28]. The combined effects of pneumoperitoneum and a steep Trendelenburg position could be synergistic [29]. The optimal PEEP of the individualized group was slightly higher than the levels of individualized PEEPs in previous studies conducted in patients undergoing open abdominal or laparoscopic surgery [13,30,31]. This may be ascribed to the combined effect of high intra-abdominal pressure and a steep Trendelenburg position, suggesting that greater PEEP would be needed for patients under this condition than for those undergoing open or laparoscopic surgery. However, increased driving pressures by pneumoperitoneum and a steep Trendelenburg position in patients undergoing robot-assisted radical prostatectomy are known to be more distributed to the chest wall rather than the lungs [32].

In a prior study, a PEEP of 7 cm H_2_O was suggested as the optimal PEEP aiming at the improvement of oxygenation without excessive airway pressure, which was much lower than the median value of individualized PEEPs in our study [33]. In that study, the level of intraoperative PaO_2_ was much higher when a PEEP of 10 cm H_2_O was provided; however, peak airway pressures increased over 40 cm H_2_O in some patients. In the present study, mechanical ventilation with individualized PEEP significantly increased peak inspiratory pressures during pneumoperitoneum compared to mechanical ventilation with a standardized PEEP of 7 cm H_2_O. However, dynamic and static compliance was significantly higher and the driving pressure was significantly lower in the individualized group than in the standardized group.

However, dynamic compliance was not significantly different between groups at T2. This may be due to the lack of sufficient time to exert the effects of individualized PEEP on the dynamic compliance. In addition, T2 was the time point immediately after the recruitment maneuver. The recruitment maneuver could have mitigated the effect of individualized PEEP in the dynamic compliance. However, the peak inspiratory pressure and the driving pressure were significantly different at T2 and T3, as shown in Figure 4 and Supplemental Table S1. The effect of individual PEEP seems to be delayed as the pneumoperitoneum lasts and atelectasis gradually develops as the laparoscopic surgery is ongoing.

Mechanical ventilation without PEEP negatively impacts patient outcomes [34,35]. A recently published expert consensus recommendation stated that the initially set fixed PEEP should be individualized thereafter [10]. However, how to individualize and determine the optimal PEEP remains unclear. Various methods to determine individualized PEEP have been reported. PEEP titrated using electrical impedance tomography reduced postoperative atelectasis [36] and improved intraoperative oxygenation [37]. Individualized PEEP determined by maximizing lung compliance, which was also used in the present study, improved oxygenation and respiratory mechanics [11,13]. In terms of the simplicity of implementation, this could be a chief and practical substitute for titrating an individualized PEEP compared to electrical impedance tomography. Another study suggested the titration of PEEP according to intra-abdominal pressure [38]. The transpulmonary driving pressure was significantly decreased by PEEP titrated to the intra-abdominal pressure compared to a fixed PEEP of 5 cm H_2_O [38]. However, the previous study did not evaluate the influence of PEEP titration on the clinical outcomes, and only three levels of PEEP according to intra-abdominal pressure were compared without individualization. Another study reported that the PEEP titration strategy based on minimizing the driving pressure shows favorable effects on clinical outcomes in thoracic surgery [39]. Our study also showed that individualized PEEP significantly decreased the driving pressure during the pneumoperitoneum. A large randomized trial testing driving-pressure-guided PEEP individualization is ongoing [40]. It is currently unclear which method of determining the optimal PEEP is better between driving-pressure-guided and dynamic-compliance-guided titration.

Vasopressor requirements and the incidence of its use were greater in the individualized group. Fluid administration was also greater in the individualized group. These results suggest that individualized PEEP may have adverse hemodynamic effects. However, a previous large multicenter study reported no significant difference in the vasopressor requirements and fluid administration [31]. As our study was not adequately powered to the hemodynamic parameters, further studies are required to evaluate the hemodynamic effect of individualized PEEP.

This study has several limitations. Firstly, we did not titrate the level of PEEP after the end of pneumoperitoneum and provided the same standardized PEEP of 7 cm H_2_O in both groups. This might have affected our results by diluting the effects of individualized PEEP during pneumoperitoneum on atelectasis. Secondly, ultrasonographic findings may be subjective and operator dependent. However, the ultrasound examination was performed by our two trained ultrasound investigators, who were completely blinded to the group allocation. This might have reduced the bias. Thirdly, since the sample size was calculated for the lung ultrasound score, this study was not sufficiently powered to detect any difference in the incidence of postoperative respiratory complications between the two groups. Fourthly, we performed the recruitment maneuver once every hour for patient safety during pneumoperitoneum. Although the last ultrasound examination was performed about 40 min after the last recruitment maneuver and this interval was similar between groups, this might have mitigated the effects of individualized PEEP on the intraoperative oxygenation. Fifthly, we did not perform additional recruitment maneuver immediately after the decremental PEEP trial, leading to the derecruitment during the decremental PEEP trial. This may have reduced the effect of individualized PEEP on atelectasis and respiratory mechanics. Sixthly, the intra-abdominal pressure, which is relevant for estimating the effect of PEEP on respiratory parameters and atelectasis, was not recorded in our study. Finally, we did not measure the lung ultrasound score after the patient’s arrival at the post-anesthesia care unit, although the ultrasound examination was performed just after extubation in the operating room. Therefore, we could not confirm whether the effects of individualized PEEP on the postoperative atelectasis persisted in the post-anesthesia care unit. However, there was no significant difference in the incidence of postoperative atelectasis evaluated by the chest X-ray taken at the post-anesthesia care unit. Previous studies have reported that the effects of an intraoperative PEEP on intraoperative oxygenation are abrogated after extubation [37]. Further study is needed to confirm whether the residual effects of individualized PEEP on atelectasis persist after extubation.

## 5. Conclusions

Mechanical ventilation with individualized PEEP determined by maximal respiratory compliance during the decremental PEEP trial significantly decreased postoperative atelectasis, evaluated using lung ultrasonography, in patients undergoing robot-assisted radical prostatectomy with pneumoperitoneum in the steep Trendelenburg position. Our results support the use of individualized PEEP in such patients. A larger randomized trial should investigate clinical outcomes such as the incidence of respiratory or surgical complications. Future studies should also discuss which method is more effective in determining the optimal individualized PEEP.

## Figures and Tables

**Table 1:** Comparison of intraoperative anesthesia-related variables between the individualized and standardized groups.

Characteristic	Individualized Group (*n* = 30)	Standardized Group (*n* = 30)	Standardized Differences	*p*-Values
Demographic data				
Age, year	64.5 ± 6.6	67.3 ± 6.4	0.419	0.131
Height, cm	166.8 ± 4.2	166.8 ± 5.7	0.019	0.951
Weight, cm	69.1 ± 10.1	70.5 ± 9.7	0.145	0.576
Body mass index, kg/m^2^	24.8 ± 3.2	25.3 ± 2.9	0.160	0.537
Baseline medical status				
Current smoker, *n*	0 (0.0)	1 (3.3)	0.263	0.999
Hypertension, *n*	14 (46.7)	11 (36.7)	0.204	0.432
Diabetes mellitus, *n*	3 (10.0)	10 (33.3)	0.591	0.028
Cardiac disease, *n*	2 (6.7)	2 (6.7)	<0.001	0.999
Neurologic disease, *n*	1 (3.3)	1 (3.3)	<0.001	0.999
Thyroid disease, *n*	1 (3.3)	1 (3.3)	<0.001	0.999
Dyslipidemia, *n*	3 (10.0)	4 (13.3)	0.104	0.999
Chronic liver disease, *n*	3 (10.0)	2 (6.7)	0.121	0.999
Preoperative laboratory findings				
Hemoglobin, g/dL	14.2 ± 1.0	14.0 ± 0.9	0.213	0.412
Hematocrit, %	42.4 ± 2.7	41.3 ± 2.6	0.385	0.141
Albumin, g/dL	4.3 (4.2–4.5)	4.4 (4.2–4.6)	0.143	0.371
Glucose, mg/dL	105.0 (98.0–117.0)	110.5 (102.0–143.0)	0.412	0.195
Serum creatinine, mg/dL	0.92 (0.82–1.02)	0.93 (0.78–1.02)	0.079	0.871
Estimated glomerular filtration rate, mL/min/1.73 m^2^	82.0 (72.8–93.9)	81.5 (72.4–97.3)	0.096	0.982
C-reactive protein, mg/dL	0.09 (0.04–0.19)	0.07 (0.04–0.13)	0.204	0.406
Prostate size, mL	37.1 (33.8–42.6)	35.7 (29.7–43.0)	0.190	0.469

Values are reported as mean ± SD or median (interquartile range) or number (%).

**Table 2:** Comparison of intraoperative anesthesia-related variables between the individualized and standardized groups.

Characteristic	Individualized Group (*n* = 30)	Standardized Group (*n* = 30)	Standardized Differences	*p*-Values
Surgeons				
A/B/C/D	5/4/7/14	0/1/8/21	0.799	0.041
Intraoperative variables				
Operation time, min	120.0 (95.0–165.0)	100.0 (90.0–125.0)	0.672	0.023
Anesthesia time, min	153.5 (130.0–210.0)	137.5 (125.0–150.0)	0.690	0.024
Duration of pneumoperitoneum, min	90.0 (75.0–140.0)	77.5 (70.0–95.0)	0.522	0.106
Administered crystalloid, mL	1270.0 ± 486.6	838.3 ± 347.8	1.021	<0.001
Dose of anesthetics				
Propofol, mg	1237.0 (941.0–1391.0)	1100.0 (1000.0–1288.0)	0.288	0.482
Remifentanil, ng	1329.9 ± 431.6	1221.9 ± 254.5	0.305	0.244
Estimated blood loss, mL	240.0 (200.0–300.0)	175.0 (100.0–300.0)	0.287	0.144
Use of vasopressor				
Total, %	27 (90)	17 (56.7)	0.800	0.004
Ephedrine, %	25 (83.3)	17 (56.7)	0.608	0.024
Ephedrine dose, mg	10.0 (5.0–20.0)	5.0 (0.0–10.0)	0.806	0.006
Phenylephrine, %	4 (13.3)	3 (10.0)	0.104	0.999
Phenylephrine dose, μg	0.0 (0.0–0.0)	0.0 (0.0–0.0)	0.215	0.701
Intraoperative desaturation, *n*	1 (3.3)	4 (13.3)	0.119	0.353

Values are reported as mean ± SD or median (interquartile range) or number (%).

**Table 3:** Comparison of lung ultrasound scores, postoperative laboratory findings, and complications between the individualized and standardized groups.

Characteristic	Individualized Group (*n* = 30)	Standardized Group (*n* = 30)	*p*-Value	Mean or Median or Risk Difference (95% Confidence Interval)
Lung ultrasound score				
Preoperative baseline	6.0 (4.0–11.0)	4.5 (3.0–9.0)	0.143	−1.0 (−4.0 to 0.0)
After extubation	8.1 ± 5.7	12.2 ± 4.2	0.002	−4.13 (−6.74 to –1.53)
Difference	−0.5 ± 2.7	6.0 ± 2.9	<0.001	−6.53 (−8.00 to –5.07)
Postoperative laboratory findings (POD #1)				
Hemoglobin, g/dL	12.8 ± 1.0	12.9 ± 1.0	0.608	−0.13 (−0.65 to 0.38)
Hematocrit, %	38.1 (37.5–39.4)	38.3 (36.3–40.0)	0.918	0.05 (–1.30 to 1.30)
Albumin, g/dL	3.7 ± 0.2	3.8 ± 0.3	0.023	−0.15 (−0.28 to −0.22)
Glucose, mg/dL	112.0 (105.0–120.0)	118.5 (106.0–129.0)	0.311	5.00 (−4.00 to 13.00)
Serum creatinine, mg/dL	0.84 (0.77–0.96)	0.87 (0.74–0.98)	0.947	0.00 (−0.08 to 0.09)
Estimated glomerular filtration rate, mL/min/1.73 m^2^	89.9 ± 18.2	89.4 ± 19.1	0.926	0.45 (−9.19 to 10.09)
C-reactive protein, mg/dL	3.6 ± 1.6	3.7 ± 1.3	0.766	−0.11 (−0.85 to 0.63)
Postoperative respiratory complications				
Total	10 (33.3)	15 (50.0)	0.190	0.50 (0.18 to 1.42)
Hypoxemia, *n*	6 (20.0)	11 (36.7)	0.152	0.43 (0.14 to 1.38)
Bronchospasm, *n*	0 (0.0)	0 (0.0)	NA	NA
Laryngospasm, *n*	0 (0.0)	0 (0.0)	NA	NA
Pneumothorax, *n*	0 (0.0)	0 (0.0)	NA	NA
Pleural effusion, *n*	0 (0.0)	0 (0.0)	NA	NA
Atelectasis, *n*	6 (20.0)	5 (16.7)	0.739	1.25 (0.34 to 4.64)
Pulmonary infiltration, *n*	0 (0.0)	2 (6.7)	0.492	0.48 (0.37 to 0.63)
Postoperative other complications				
Total	2 (6.7)	3 (10.0)	0.999	0.64 (0.10 to 4.15)
Anastomosis site leakage, *n*	1 (3.3)	1 (3.3)		
Acute kidney injury, *n*	1 (3.3)	1 (3.3)		
Variant angina, *n*	0 (0.0)	1 (3.3)		
Subcutaneous emphysema, *n*	2 (6.7)	3 (10.0)	0.999	0.64 (0.10 to 4.15)
Length of hospital stay, day	5.0 (5.0–5.0)	5.0 (5.0–5.0)	0.410	0.00 (0.00 to 0.00)
ICU admission, *n*	0 (0.0)	0 (0.0)	NA	NA

Values are reported as mean ± SD or median (interquartile range) or number (%). POD = postoperative day; NA = not available; ICU = intensive care unit.

## Data Availability

The data presented in this study are available on request from the corresponding author.

## Supplementary Materials

The following are available online at https://www.mdpi.com/2077-0383/10/4/850/s1, Table S1: Comparison of ventilator-related parameters and intraoperative arterial blood gas analysis between the individualized and standardized groups.
