# Supplementary material for: The Effect of Ventilation with Individualized Positive End-Expiratory Pressure on Postoperative Atelectasis in Patients Undergoing Robot-Assisted Radical Prostatectomy: A Randomized Controlled Trial
Source: J Clin Med. 2021 Feb 19;10(4):850. doi: 10.3390/jcm10040850 (PMC7922101; doi:10.3390/jcm10040850)
Supplement: Supplementary file 1 [file jcm-10-00850-s001.pdf]

Supplementary Material

Supplementary Table S1. Comparison of ventilator-related parameters and intraoperative arterial blood gas analysis between the individualized and standardized groups.

**Characteristic**	**Individualized Group (n = 30)**				**Standardized Group (n = 30)**				***p*-Value**			
	T1	T2	T3	T4	T1	T2	T3	T4	T1	T2	T3	T4
Tidal volume, mL	378.5 ± 35.0	389.1 ± 29.0	389.6 ± 31.0	386.1 ± 32.5	379.7 ± 39.1	392.5 ± 42.3	386.9 ± 42.0	380.0 ± 38.6	0.898	0.713	0.781	0.511
PEEP, cm H_2O	7 (7.0–7.0)	14.0 (12.0–18.0)	14.0 (12.0–18.0)	7 (7.0–7.0)	7 (7.0–7.0)	7 (7.0–7.0)	7 (7.0–7.0)	7 (7.0–7.0)	NA	<0.001	<0.001	NA
PIP, cm H_2O	16.1 ± 2.3	32.4 ± 4.0	32.7 ± 3.8	17.4 ± 1.9	15.3 ± 2.0	26.4 ± 3.8	27.7 ± 3.3	16.8 ± 2.1	0.133	<0.001	<0.001	0.256
Pplt, cm H_2O	13.5 (13.0–14.0)	28.5 (26.0–33.0)	29.0 (27.5–32.5)	15.0 (14.0–16.0)	14.0 (13.0–15.5)	25.0 (22.5–p26.5)	26.0 (24.0–27.0)	15.0 (14.0–16.0)	0.197	<0.001	<0.001	0.754
Dynamic compliance, mL/cm H_2O	43.5 ± 10.0	22.6 ± 4.0	22.3 ± 4.3	38.3 ± 7.0	47.6 ± 9.8	20.9 ± 3.9	19.1 ± 3.5	40.4 ± 8.8	0.110	0.086	0.003	0.312
Static compliance, mL/cm H_2O	58.9 ± 14.5	26.6 ± 5.7	25.8 ± 4.8	49.8 ± 8.5	52.6 ± 12.1	21.5 ± 3.6	19.9 ± 3.4	47.2 ± 11.3	0.130	0.001	<0.001	0.402
Driving pressure, cm H_2O	6.5 (6.0–7.0)	15.0 (13.0–17.5)	15.0 (13.0–17.5)	8.0 (7.0–9.0)	7.0 (6.0–8.5)	18.0 (15.5–19.5)	19.0 (17.0–20.0)	8.0 (7.0–9.0)	0.197	0.004	<0.001	0.754
PaO_2/FiO_2	503.7 ± 136.8	363.2 ± 82.8	369.8 ± 77.5	429.2 ± 91.4	504.5 ± 197.0	321.5 ± 114.1	341.6 ± 108.5	356.5 ± 100.7	0.985	0.111	0.252	0.005
$PaCO_2$, mmHg	43.1 ± 3.9	42.0 (40.0–44.0)	48.5 (44.0–52.0)	46.2 ± 6.1	42.9 ± 4.1	44.0 (40.0–48.0)	47.5 (44.0–52.0)	49.1 ± 6.8	0.873	0.041	0.941	0.087
pH	7.42 (7.41–7.43)	7.42 ± 0.04	7.37 ± 0.04	7.39 (7.36–7.43)	7.41 (7.39–7.44)	7.40 ± 0.04	7.36 ± 0.05	7.36 (7.33–7.39)	0.498	0.021	0.372	0.032

HCO_3^-, mmol/L	27.9 (27.2–28.5)	27.5 (26.6–28.5)	27.8 (26.6–28.5)	27.4 ± 1.9	27.6 (26.6–28.5)	27.2 (26.0–28.2)	27.2 (26.3–28.2)	27.5 ± 1.4	0.170	0.371	0.143	0.875
SaO_2, %	100 (99–100)	99 (99–100)	99 (99–100)	100 (99–100)	100 (99–100)	99 (98–99)	99 (98–100)	99 (98–100)	0.323	0.042	0.256	0.173

Values are reported as mean ± SD or median (interquartile range). PEEP = positive end-expiratory pressure; PIP = peak inspiratory pressure, Pplt = plateau pressure. Plateau pressures were measured only in 44 patients (24 patients in individualized group and 20 patients in standardized group). T1= after anesthesia induction, T2 = after achieving the pneumoperitoneum under steep Trendelenburg position, T3 = 1 h after T2, and T4 = just before extubation in the operating room.
